# The Impact of Prehabilitation on Patient Outcomes in Oesophagogastric Cancer Surgery: Combined Data from Four Prospective Clinical Trials Performed Across the UK and Ireland

**DOI:** 10.3390/cancers17111836

**Published:** 2025-05-30

**Authors:** Sowrav Barman, Beth Russell, Robert C. Walker, William Knight, Cara Baker, Mark Kelly, James Gossage, Janine Zylstra, Greg Whyte, James Pate, Jesper Lagergren, Mieke Van Hemelrijck, Mike Browning, Sophie Allen, Shaun R. Preston, Javed Sultan, Pritam Singh, Timothy Rockall, William B. Robb, Roisin Tully, Lisa Loughney, Jarlath Bolger, Jan Sorensen, Chris G. Collins, Paul A. Carroll, Claire M. Timon, Mayilone Arumugasamy, Thomas Murphy, Noel McCaffrey, Mike Grocott, Sandy Jack, Denny Z. H. Levett, Tim J. Underwood, Malcolm A. West, Andrew R. Davies

**Affiliations:** 1 Guy’s & St Thomas’ Oesophago-Gastric Centre, London SE1 7EH, UK; william.r.knight@googlemail.com (W.K.); cara.baker@gstt.nhs.uk (C.B.); mark.kelly@gstt.nhs.uk (M.K.); james.gossage@gstt.nhs.uk (J.G.); janinezylstra@nhs.net (J.Z.); 2 School of Cancer & Pharmaceutical Sciences, King’s College London, London WC2R 2LS, UK; jesper.lagergren@kcl.ac.uk; 3 Transforming Cancer OUtcomes Through Research (TOUR), School of Cancer & Pharmaceutical Sciences, King’s College London, London WC2R 2LS, UK; beth.russell5@nhs.net (B.R.); mieke.vanhemelrijck@kcl.ac.uk (M.V.H.); 4 School of Cancer Sciences, Faculty of Medicine, University of Southampton, Southampton SO16 6YD, UK; r.c.walker@soton.ac.uk (R.C.W.); t.j.underwood@soton.ac.uk (T.J.U.); m.west@soton.ac.uk (M.A.W.); 5 Royal Surrey NHS Foundation Trust, Guildford GU2 7XX, UK; sophieallen@doctors.org.uk (S.A.); shaun.preston@nhs.net (S.R.P.); p.singh9@nhs.net (P.S.); t.rockall@nhs.net (T.R.); 6 Research Institute for Sport and Exercise Sciences (RISES), Liverpool John Moores University, Liverpool L3 3AF, UK; gregwhyte27@icloud.com; 7 Marylebone Health Group, London W1G 7HH, UK; jamespate@marylebonehealthgroup.com; 8 Department of Molecular Medicine and Surgery, Karolinska University Hospital, Karolinska Institutet, SE 17176 Stockholm, Sweden; 9 Maidstone and Tunbridge Wells NHS Trust, Maidstone ME16 9QQ, UK; mbrowning@nhs.net; 10 Faculty of Health and Medical Sciences, School of Medicine, University of Surrey, Guildford GU2 7AL, UK; 11 Manchester Academic Health Science Centre, University of Manchester, Manchester M13 9PL, UK; javed.sultan@nca.nhs.uk; 12 Department of Oesophago-Gastric & Bariatric Surgery, Salford Royal Hospital, Northern Care Alliance NHS Foundation Trust, Salford M6 8HD, UK; 13 Department of Upper GI Surgery, Beaumont Hospital, D09 V2N0 Dublin, Ireland; robb.will@gmail.com (W.B.R.); roisintully@rcsi.ie (R.T.); jarbolger@rcsi.ie (J.B.); marumugasamy@rcsi.ie (M.A.); 14 Department of Surgery, Royal College of Surgeons in Ireland, St. Stephen’s Green, D02 YN77 Dublin, Ireland; lisa.loughney@gmail.com (L.L.); jansorensen@rcsi.ie (J.S.); 15 ExWell Medical, c/o Irish Wheelchair Association, Blackheath Drive, D03 AW62 Dublin, Ireland; noel.mccaff@gmail.com; 16 Department of Surgery, University Hospital Galway, H91 YR71 Galway, Ireland; chris.collins@hse.ie; 17 Department of Surgery, Sligo University Hospital, F91 H684 Sligo, Ireland; paulcarroll@rcsi.ie; 18 School of Population Health, RCSI University of Medicine and Health Sciences, D09 YD60 Dublin, Ireland; clairetimon@rcsi.com; 19 Department of Surgery, Mercy University Hospital, T12 WE28 Cork, Ireland; tmurphy@muh.ie; 20 NIHR Southampton Biomedical Research Centre, Perioperative and Critical Care Theme, University Hospital Southampton NHS Foundation Trust, Southampton SO16 7QF, UK; mike.grocott@soton.ac.uk (M.G.); s.jack@soton.ac.uk (S.J.); d.levett@soton.ac.uk (D.Z.H.L.); 21 Integrative Physiology and Critical Illness Group, School of Clinical and Experimental Sciences, University of Southampton, Southampton SO17 1BJ, UK

**Keywords:** prehabilitation, oesophagogastric cancer, chemotherapy, postoperative complications, cardiorespiratory fitness

## Abstract

Background: Prehabilitation is increasingly being used in patients undergoing multimodality treatment for oesophagogastric cancer (OGC). Most studies to date have been small, single-centre trials. This collaborative study sought to assess the overall impact of prehabilitation on patient outcomes following OGC surgery. Methods: Data came from four prospective prehabilitation trials conducted in the UK or Ireland in patients undergoing multimodality treatment for OGC. The studies included three randomised and one non-randomised clinical trial, each comparing a prehabilitation intervention group to controls. The prehabilitation interventions included aerobic training delivered by exercise physiologists alongside dietetic input throughout the treatment pathway. The primary outcome was survival (all-cause and disease-specific mortality). Secondary outcomes were differences in complications, cardio-respiratory fitness (changes in VO_2 peak_ and anaerobic threshold (AT)), chemotherapy completion rates, hospital length of stay, changes in body mass index, tumour regression and complication rates of anastomotic leak and pneumonia. Cox and logistic regression analysis provided hazard ratios (HR) and odds ratios (OR), respectively, with 95% confidence intervals (CI), adjusted for confounders. Results: Among 165 patients included, 88 patients were in the prehabilitation group and 77 patients were in the control group. All-cause and disease-specific mortality were not improved by prehabilitation (HR 0.67 95% CI 0.21–2.12 and HR 0.82 95% CI 0.42–1.57, respectively). The prehabilitation group experienced fewer major complications (20% vs. 36%, *p* = 0.034; adjusted OR of 0.54; 95%CI 0.26–1.13). There was a mitigated decline in VO_2 peak_ following neo-adjuvant therapy (delta prehabilitation −1.07 mL/kg/min vs. control −2.74 mL/kg/min; *p* = 0.035) and chemotherapy completion rates were significantly higher following prehabilitation (90% vs. 73%; *p* = 0.016). Hospital length of stay (10 vs. 12 days, *p* = 0.402) and neoadjuvant chemotherapy response (Mandard 1–3 41% vs. 35%; *p* = 0.494) favoured prehabilitation, albeit not statistically significantly. Conclusion: Despite some limitations in terms of heterogeneity of study methodology, this study suggests a number of meaningful clinical benefits from prehabilitation before surgery for OGC patients. Current initiatives to agree on national standards for delivering prehabilitation and the results of ongoing trials will help to further refine this important intervention and expand the evidence base to support the widespread adoption and implementation of prehabilitation programs.

## Simple Summary

This study examined the effects of prehabilitation—structured exercise, nutrition and psychological support before surgery—on patients with oesophagogastric cancer undergoing neoadjuvant therapy and surgery. Although prehabilitation did not result in improved survival rates, it provided several meaningful benefits. Patients had fewer major complications after surgery, were better able to maintain their fitness levels during treatment and were more likely to complete their chemotherapy. These results suggest that prehabilitation can help patients better manage the physical demands of cancer treatment. Although more research is needed to establish consistent national standards, this study supports the growing use of prehabilitation in cancer care.

## 1. Introduction

Despite recent advances in surgical techniques, anaesthesia and critical care, surgery for oesophagogastric cancer (OGC) is still associated with considerable post-operative morbidity [1]. Up to 60% of patients develop post-operative complications, which contribute to a prolonged length of hospital stay, delayed recovery, increased healthcare costs, long-term disability and worse overall survival [2]. Management of OGC patients, who are often elderly and comorbid with poor functional reserve, is a clinical challenge [3,4]. Neoadjuvant therapy (NAT) combined with surgery is the treatment of choice for locally advanced oesophagogastric cancer based on a survival benefit in randomised controlled trials [5,6,7]. However, chemotherapy reduces the functional capacity and overall fitness of this already vulnerable cancer patient group before surgery [8].

Prehabilitation is a multimodal process that aims to prepare patients to withstand the negative effects of treatments such as surgery and chemotherapy. Numerous definitions of prehabilitation exist [9,10]. It is increasingly being used in patients undergoing treatment for OGC. Recent studies have suggested that prehabilitation can reduce sarcopenia, maintain fitness (e.g., VO_2_ peak) and improve quality of life in patients undergoing OGC resection [11]. However, most research to date has consisted of small, single-centre studies with low statistical power. The results of larger randomised trials are still awaited. Significant questions remain regarding which outcome measures might improve with prehabilitation, the exact nature of the intervention and the mechanisms behind any observed benefit.

With societal (Association of Upper GI Surgeons of Great Britain & Ireland—AUGIS) support, this collaborative study sought to combine data from four prospective clinical prehabilitation trials performed in the UK or Ireland to assess the overall impact on patient outcomes following OGC surgery. The primary aim of the study was to compare the effects of a structured prehabilitation program on overall survival (OS) and disease-free survival (DFS) in OGC patients undergoing neoadjuvant therapy prior to surgery. Secondary aims were to evaluate the impact of prehabilitation on complications, cardiorespiratory fitness, chemotherapy completion, length of hospital stay, changes in body composition, tumor regression, and specific complications.

## 2. Materials and Methods

A UK society (AUGIS) endorsed initiative sought to establish guidance on prehabilitation for OGC patients. As part of this peri-operative quality initiative (POQI) modified Delphi process, a contemporaneous review of the prehabilitation literature was performed and combined data from four prospective UK or Irish trials were made available to participants [12,13].

Of the four prospective clinical trials included, 3 were randomised (Southampton, Guildford, Dublin) and one was non-randomised (London). Recruitment for the studies took place between 2016 and 2020: London (2016–2020), Southampton (2016–2017), Guildford (2016–2018), and Dublin (2019–2020). All four studies compared a structured prehabilitation program (intervention) versus conventional best practice (control) in patients undergoing neoadjuvant therapy (chemotherapy or chemo-radiotherapy) for operable adenocarcinoma of the oesophagus or stomach. Ethical approval was granted for each individual study and the host institution held ethical approval for the analysis of pseudo-anonymised multi-centre data (Research Ethics Committee reference numbers 16/SC/0438, 15/SC/078, 16/LO/1702, Beaumont 18/58, ECM 4 (mm) 19/04/19, DCUREC/2018/255, C.A. 2160).

All patients were investigated and managed according to similar staging and treatment pathways, overseen by a multidisciplinary team (MDT) meeting. Table 1 summarises the similarities and differences between the studies.

**Table 1:** Comparison of prehabilitation interventions and outcome measures across the four clinical trials.

	London	Southampton	Guildford	Dublin
Randomised	No	Yes	Yes	Yes
Duration	Diagnosis to adjuvant chemo (26 weeks)	Diagnosis to post-op (15+/−2 weeks)	Diagnosis to post-op (15 weeks)	Diagnosis to post-op (18+/−2.4 weeks)
Aerobic training	Yes *	Yes *	Yes *	Yes *
Resistance training	Yes Whole body resistance band circuit (targeting 15–20 reps) involving 12 exercises and repeat 3 times.	No	Yes 12 repetitions of 2 sets of exercises using free weights and resistance bands involving six major muscle groups.	Yes Circuit of six to ten stations for alternating upper and lower body exercises.
Dietetic input	Yes—both groups	Yes—both groups	Yes—both groups	Yes—both groups
Psychological input	Yes—as required	No	Yes—prehab only	No
Fitness assessment	CPET	CPET	CPET	6 MWT
Face-to-face/remote/hybrid	Hybrid	Face-to-face	Hybrid	Hybrid
Standard/personal	Personalised Need-based and used FITT criteria	Personalised Need-based and used FITT criteria	Personalised Need-based and used FITT criteria	Personalised Need-based and used FITT criteria
Delivered by	Exercise physiologist	Exercise physiologist	Exercise physiologist	Exercise physiologist
Outcome measures	1. Fitness (CPET—AT, VO_2_ peak, physical activity) 2. Complications 3. Chemotherapy completion and toxicity 4. Body composition (skeletal muscle mass) 5. HRQL 6. Length of stay 7. Tumour regression	1. Fitness (CPET—AT, VO_2 peak_, physical activity) 2. Complications 3. Chemotherapy completion and toxicity. 4. Sarcopenia 5. Disability adjusted survival (WHODAS) 6. Tumour regression 7. Overall survival.	1. Fitness (CPET—AT, VO_2 peak_, weekly step count, hand grip strength) 2. Complications 3. Chemotherapy completion and toxicity 4. Body composition (skeletal muscle mass) 5. Insulin resistance 6. HRQL 7. Length of stay 8. Tumour regression.	1. Fitness (6 MWT, sit-to-stand, Handgrip, Physical Activity) 2. Complications, Post-Operative Morbidity and Pathological Data. 3. Chemotherapy treatment completion, rates, Toxicity, tolerance. 4. BMI 5. HRQL (LOT-R, EQ-5D-5L, FACT-E)

CPET cardiopulmonary exercise test, 6 MWT 6 min walk test, AT anaerobic Threshold, HRQL health-related quality of life, QOL quality of life, WHODAS WHO Disability Assessment Schedule, BMI body mass index, LOT-R Life Orientation Test-Revised, FACT Functional Assessment of Cancer Therapy. * Dose and prescription of exercise intervention described separately in Table 2.

**Table 2:** Dose and prescription of exercise interventions using FITT principle.

	London	Southampton	Guildford	Dublin
Frequency	5 exercise sessions per week	3 exercise sessions per week (2 sessions per week if on cancer therapies)	2 exercise session per week (supervised) 3 exercise sessions per week (home)	3 exercise sessions per week (2–3 exercise sessions per week if on cancer therapies)
Intensity	Moderate to high	Moderate to high	Moderate to high	Interval (moderate to high); continuous (moderate)
Time	5 × 30 min sessions per week	3 × 40 min sessions per week or 2 × 30 min sessions per week if on cancer therapies	5 × 60 min sessions per week	Pre-op: First interval and continuous exercise session is 30 min duration. Second and subsequent sessions are 40 min in duration. Post-op: Initially for 20 min sessions and increase the duration of exercise by 10 min per week.
Type	Walking program with some days steady and some days with intervals.	Sitting on the stationary bicycle and pedalling at a cadence of 60–65 revolutions per minute.	5 min warm-up followed by 25 min cycling	Centre-based: upright cycle ergometer, recumbent cycle ergometer, treadmill, elliptical ergometer, and rowing ergometer, depending on patient preference. Home-based: combination of walking, jogging or cycling
Volume	150-min per week for 26 weeks	60–120 min per week for 15+/−2 weeks	300 min per week for 15 weeks	120 min per week or 80–120 min per week if on cancer therapies for 18 +/−2.4 weeks.

### 2.1. Prehabilitation Program (Exposure)

In all studies, the prehabilitation program started at the time of cancer diagnosis and continued before, during and after neoadjuvant therapy prior to surgery. The prehabilitation programmes were personalised to each individual’s physical conditioning and used established FITT principles to structure the exercise dose and prescription. The programmes were mostly hybrid (with a combination of face-to-face and remote exercise prescribed in three centres) and involved aerobic training delivered by exercise physiologists and dietitian input in all studies. Three studies incorporated resistance training into the prehabilitation intervention. Psychological input was available for selected patients in one study and for the intervention group only in another. Three out of the four studies used cardiopulmonary Exercise Testing (CPET) for fitness assessment, while one study used the 6 min walk test. The baseline body mass index (BMI) and (where available) maximum oxygen uptake (VO_2 peak_) and anaerobic threshold (AT) were measured before the intervention and repeated after completion of neo-adjuvant therapy.

Details on dose and prescription of exercise intervention using FITT principles [14,15] are described in the table below (Table 2):

### 2.2. Combined Study Outcomes

The primary outcome measure was survival (all-cause and disease-specific mortality) comparing prehabilitation and control groups from the combined database. Secondary outcomes were major complication rates (Clavien–Dindo classification), changes in cardio-respiratory fitness during treatment (changes in VO_2 peak_ and anaerobic threshold), chemotherapy completion rates, hospital length of stay, body composition/body mass index (BMI), tumour regression (Mandard tumour regression grade [MTRG]) and specific complication rates (anastomotic leak and pneumonia) as defined by the esophageal cancer complications group (ECCG) criteria [16].

All-cause mortality was calculated using date of surgery to date of death or date last seen, if still alive. Similarly, disease-specific mortality was calculated using date of surgery to date of confirmed recurrence (radiological or histological) or date last seen (if no recurrence). Patients were followed up for a median duration of 31 months (IQR: 20–43 months) from the date of surgery. Complications were classified as none or minor (0–2) or major (3–4) according to the Clavien–Dindo grading system [17]. A successful BMI outcome was defined as maintenance of a healthy BMI, or a positive move towards a healthy BMI comparing baseline to post-NAT (i.e., overweight to healthy, obese to overweight, underweight to healthy etc.). For tumour regression, patients were classified as either responders (MTRG 1–3) or non-responders (MTRG 4–5).

### 2.3. Statistical Analysis

Descriptive statistics were used for the baseline characteristics, stratified by each centre and as a combined dataset, dividing patients into prehabilitation and control groups. Categorical variables were assessed using the chi-squared test.

Cox proportional hazards regression models were used to assess the association between the prehabilitation intervention and time to death or time to recurrence. Unadjusted and adjusted analyses were performed, the latter adjusting for age (continuous), sex (male or female), ASA (1–2 or 3–4), tumour stage (cTNM—T0–2/T3–4 or N0/N1–3), baseline BMI (healthy, overweight, or obese) and neo-adjuvant therapy (NAC or NACRT). Direct acyclic graphs were used to identify the adjustments required for each model. Each study’s data was first analysed individually before results were meta-analysed using a random effects model to generate a pooled hazard ratio (HR) and 95% confidence interval (CI).

For post-operative complications, Firth’s logistic regression models were applied to assess the association between the exercise intervention and the outcome (Clavien–Dindo 3–4 complication). This method was used to account for the small sample size and separation in the data. Models were adjusted for age, sex, ASA and NAC/NACRT to generate odds ratios (OR) and 95% CI. Pooled ORs were then calculated using random effects meta-analysis models. Changes in fitness prior to and following neo-adjuvant therapy were assessed using VO_2 peak_ and AT. The difference in VO_2 peak_ and AT values for the exercise intervention and control groups pre- and post-NAT were compared using the Student’s *t*-test.

## 3. Results

### 3.1. Patient Characteristics

Table 3 provides an overview of the study and patient characteristics. The four trials included 165 participants, of whom 88 underwent prehabilitation (intervention) and 77 received conventional care (controls).

### 3.2. Survival

**Figure 1:**
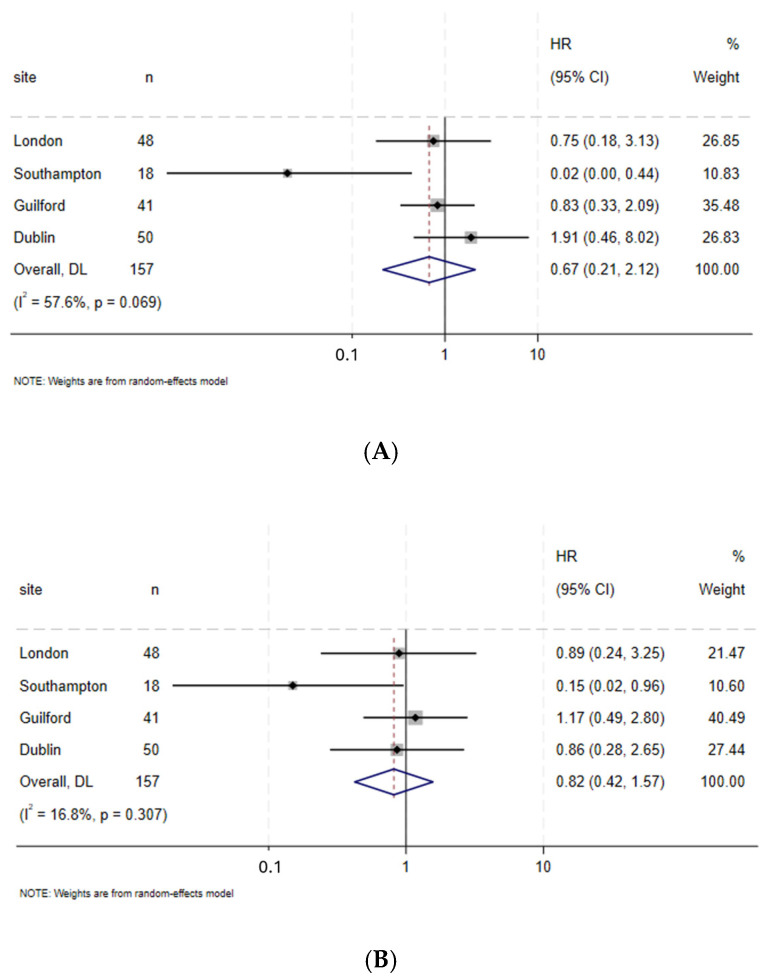
(**A**) Forest plot for overall survival after neoadjuvant therapy followed by surgery. (**B**) Forest plot for disease-free survival after neoadjuvant therapy followed by surgery. Adjusted for age, sex, ASA, baseline BMI, T and N stage, NAC/NACRT.

In the pooled analysis, there was no significant difference in all-cause or disease-specific mortality between the exercise and control groups, although the point estimates favoured the intervention group (OS; HR = 0.67, 95% CI 0.21–2.12 and DFS; HR = 0.82, 95% CI 0.42–1.57, respectively) (Figure 1A,B).

### 3.3. Complications

**Figure 2:**
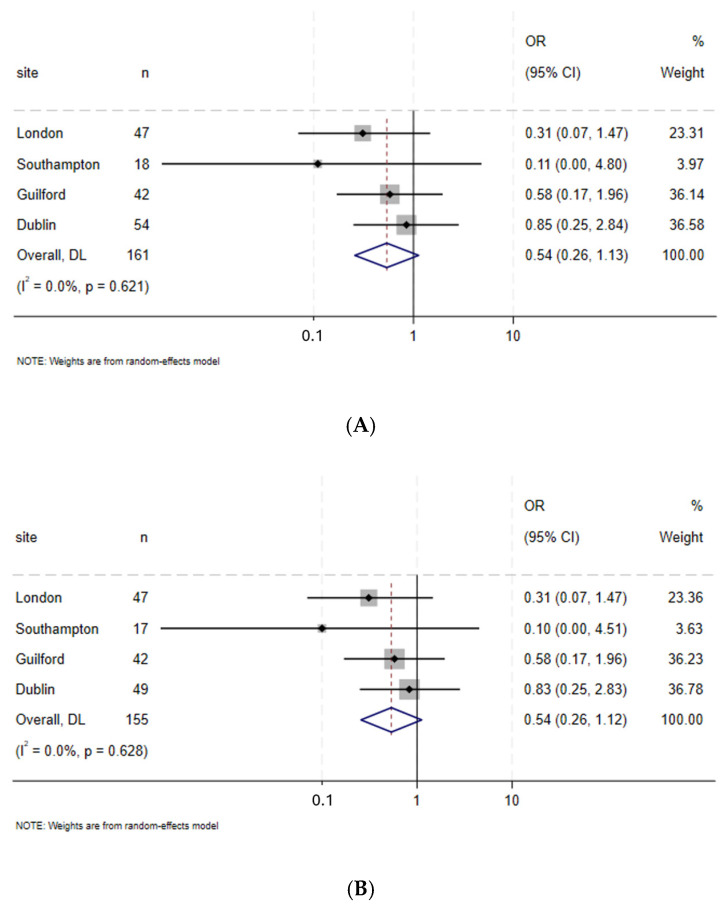
(**A**). Forest plot based on Clavien–Dindo (0–2 vs. 3–4) in both groups—prehabilitation vs. control; all patients. (**B**) Forest plot based on Clavien–Dindo (0–2 vs. 3–4) in both groups—Prehabilitation vs. control, excluding distal gastrectomy.

The prehabilitation group had fewer major complications in overall and sub-group analysis (excluding distal gastrectomy patients) (Clavien–Dindo 3–4 overall: prehabilitation 20% vs. control 36% (*p* = 0.034); sub-group analysis: prehabilitation 21% vs. control 37% (*p* = 0.032)). On multivariable analysis, the pooled ORs favoured prehabilitation although this did not reach statistical significance (OR overall: 0.54 95%CI 0.26–1.13 and OR sub-group: 0.54 95% CI 0.26–1.12) (Figure 2A,B).

### 3.4. Fitness

In combined data of the three studies with data available, changes in VO_2 peak_ before and after NAT showed a significantly mitigated decline in the prehabilitation group (prehabilitation −1.07; control −2.74, *p* = 0.035) (Table 4). For anaerobic threshold this also favoured prehabilitation, albeit not reaching statistical significance (prehabilitation −0.96; control −1.78; *p* = 0.385) (Table 5).

### 3.5. Chemotherapy Completion

Patients in the prehabilitation group had a significantly higher rate of completing all prescribed neo-adjuvant chemotherapy (prehabilitation 79/88 (90%) vs. control 56/74 (73%); *p* = 0.016).

### 3.6. Length of Hospital Stay

**Figure 3:**
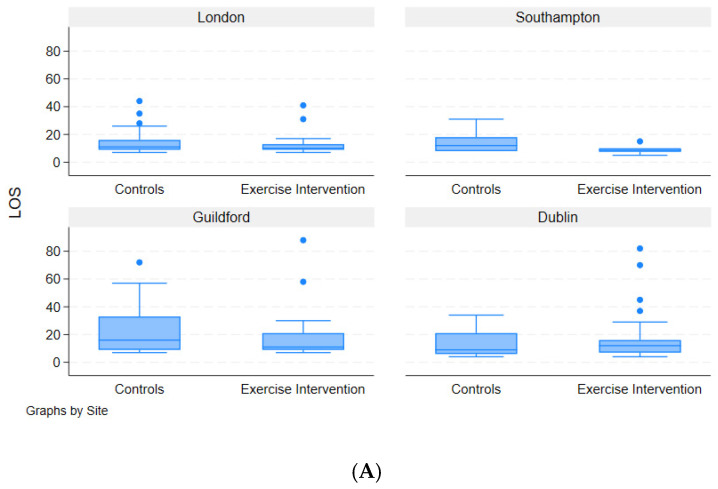

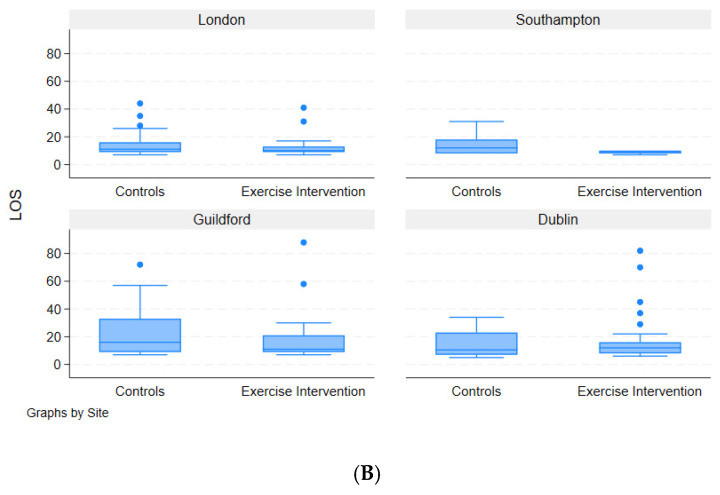
(**A**). Box plot length of stay; inclusive of all surgery types. (**B**) Box plot length of stay; excluding distal gastrectomy.

Overall length of hospital stay seemed to be lower in the prehabilitation group across all patients (median 10 days vs. 11 days; *p* = 0.377) and in sub-group analysis (excluding distal gastrectomy) (median 10 days vs. 12 days; *p* = 0.402), although neither reached statistical significance. *p*-value for *t*-test (all sites combined) = 0.377 (Figure 3A,B).

### 3.7. Changes in Body Mass Index

**Figure 4:**
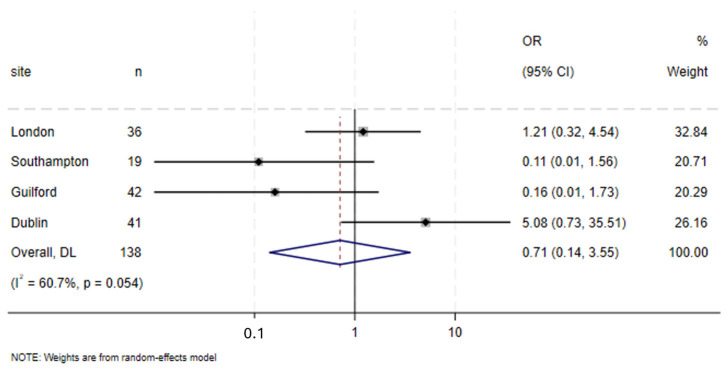
Forest plot of positive changes in BMI (adjusted for age, sex, baseline BMI, cT and cN stage).

There was no overall difference between the groups in terms of a shift towards a healthier BMI during NAT (OR = 0.71, 95% CI 0.14–3.55, I2 = 60.7%, *p* = 0.054) (Figure 4).

### 3.8. Tumour Regression

Overall, more patients exhibited tumour regression in the prehabilitation group (Mandard 1–3 responder 56% vs. 45% *p* = 0.2105). However, there were differences between the groups with more use of NACRT but less use of FLOT in the prehabilitation group. Therefore, analysis was also stratified by NAT. In NAC patients, there were more responders in the prehabilitation group (Mandard 1–3 prehabilitation 41% vs. control 35% *p* = 0.494) albeit not statistically significant, despite a greater proportion of patients receiving FLOT chemotherapy in the control group. In NACRT patients, response rates were similar between groups (Mandard 1–3 prehabilitation 82% vs. control 79% *p* = 0.800).

### 3.9. Specific Complications

There were no statistically significant differences between groups for anastomotic leak in all patients (prehabilitation 6% vs. control 8%; *p* = 0.757) and in sub-group analysis excluding distal gastrectomy patients (prehabilitation 6% vs. control 8%; *p* = 0.429).

There were no statistically significant differences between groups for pneumonia in all patients (prehabilitation 16% vs. control 20%; *p* = 0.682) and in sub-group analysis excluding distal gastrectomy patients (prehabilitation 18% vs. control 20%; *p* = 0.379).

## 4. Discussion

This study, utilising combined data from four prospective UK prehabilitation trials in OGC patients, has shown certain benefits for patients receiving a prehabilitation intervention [11,18,19]. Whilst the primary outcome measures of overall and disease-free survival were not significantly different between the groups, the point estimates favoured prehabilitation over conventional best practice. There were fewer severe complications and a mitigated decline in cardio-respiratory fitness through neo-adjuvant therapy in the prehabilitation group. Chemotherapy completion rates were also improved.

Several methodological issues warrant further discussion. The background of the study was to provide contemporaneous data for a society-endorsed peri-operative quality initiative process aiming to provide guidance on prehabilitation in OGC patients [13]. As part of this process, a systematic review of the literature was performed and combined unit data from four UK-based OGC prehabilitation trials were presented to participants [12]. Strengths of the combined analysis include the multi-centre nature (increasing the external validity and generalisability of the findings) and a larger sample size, allowing for more detailed statistical analysis with greater power to adjust for confounders. However, it is acknowledged that, despite this approach, the sample size remains a limitation, particularly for subgroup analyses. The introduction of heterogeneity was also a limitation. Whilst three randomised controlled trials (RCTs) were among the four studies included, the other study was a non-randomised clinical trial, inherently more susceptible to selection bias. As a result, outcome measures were adjusted for confounders during analysis. Each site was analysed individually before performing a pooled analysis. To address this further, sensitivity analyses were conducted, excluding one centre at a time. Notably, excluding the non-randomised cohort did not alter the overall results. There was inevitably a degree of heterogeneity in the prehabilitation programs, which may have led to variability in the quality of the intervention. Some variation in the neo-adjuvant treatment strategies (NAC and NACRT) employed was also observed, reflecting practice at the time. Different fitness measurement tools were used across the studies with three of the four studies employing cardiopulmonary exercise testing (CPET) and one study using the 6 min walk test. Stratifying analyses to accommodate such differences inevitably reduced the statistical power.

Numerous studies have assessed the role of prehabilitation across a range of tumour groups [20,21,22,23,24]. None have demonstrated a clear overall survival benefit, although a large population-based study from the United States showed improved survival in fitter patients undergoing cancer treatment with a 25% reduction in all-cause mortality [25]. One UK study has shown worse survival in OGC patients declining to participate or dropping out of a prehabilitation programme, highlighting the importance of accessing the highest risk patients for these interventions [26]. The OptiTrain trial investigated a 16-week high-intensity interval training (HIIT) program alongside either aerobic training (AT-HITT) or resistance training (RT-HITT) compared to standard care during chemotherapy for breast cancer showing significantly improved overall survival for prehabilitation compared to usual care [27]. A systematic review found a decrease in the incidence of complications after OGC treatment defined as Clavien–Dindo grade 2 or higher and a lower incidence of pneumonia in patients receiving prehabilitation [28]. The BEAUTY study found an increase in VO_2 peak_ in the exercise group and an improvement of around 1 min in the submaximal treadmill test at 24 weeks [29]. The PREPARE trial, which focused on an OGC population, reported a reduction in postoperative pneumonia rates from 66% to 26% and a decrease in the median length of hospital stay from 13 to 10 days [30]. A meta-analysis found that prehabilitation reduced hospital length of stay by nearly two days versus standard care across various surgical specialties [31]. A number of studies have assessed histological response to neo-adjuvant therapy, showing improved response in exercising patients [18]. A study in breast cancer patients found that exercise led to improved tumour regression, with a higher likelihood of complete response compared to usual care [32]. Similarly, a study in rectal cancer showed significantly enhanced tumour regression after NACRT and surgery in the prehabilitation group [33].

Pending the reporting of large ongoing randomised trials [34,35], there remain considerable uncertainties with regard to the specific aspects of prehabilitation interventions that afford the greatest benefit. A national survey of prehabilitation practice in the UK highlighted significant barriers to widespread adoption, most notably financial support and staffing. Variations in the location and personnel delivering exercise interventions were conspicuous, as were inconsistencies in the availability of the other facets of multimodal prehabilitation such as psychological support [36]. The resultant societal guidance has acknowledged these uncertainties whilst concluding that the evidence in support of prehabilitation is now sufficiently strong that it be recommended as standard of care in patients undergoing treatment for OGC [13].

## 5. Conclusions

In conclusion, despite some limitations in terms of heterogeneity of study methodology and prehabilitation interventions, this study has indicated a number of benefits of prehabilitation in patients who undergo neoadjuvant therapy and surgery for oesophagogastric cancer. There was no statistically significant difference in survival.

## Figures and Tables

**Table 3:** Patient characteristics and summary of outcomes.

All Sites Combined					
	Prehabilitation Group (N = 88)		Control Group (N = 77)		*p*-Value
	*n*	%	*n*	%	
Age, years (mean (SD))	63.25 (9.34)		61.69 (8.50)		0.269 b
Sex					
Male	69	78.4	63	81.8	0.585 a
Female	19	21.6	14	18.2	
Baseline BMI, kg/m^2^ (mean (SD))					
Underweight	0	0	0	0	0.756 a
Healthy	27	30.7	23	29.9	
Overweight	32	36.4	22	28.6	
Obese	24	27.3	22	28.6	
Missing	5	5.7	10	13	
Post-NAT BMI, kg/m^2^ (mean (SD))					
Underweight	1	1.1	0	0	0.818 a
Healthy	27	30.7	22	28.6	
Overweight	31	35.2	28	36.4	
Obese	17	19.3	14	18.2	
Missing	12	13.6	13	16.9	
VO_2 peak_, mean (SD), mL/kg/min					
At baseline	22.19 (5.54)		22.18 (3.89)		
Post-neoadjuvant treatment	20.92 (3.99)		19.23 (3.25)		
Delta/change in VO_2 peak_	−1.07 (4.47)		−2.74 (2.91)		0.035 b
AT, mean (SD), mL/kg/min					
At baseline	14.04 (3.69)		14.48 (3.24)		
Post-neoadjuvant treatment	12.81 (2.62)		11.81 (2.68)		
Delta/change in anaerobic threshold	−0.96 (4.00)		−1.78 (5.72)		0.385 b
ASA					
2	58	65.9	51	66.2	0.965 a
3	30	34.1	26	33.8	
Treatment characteristics					
Neoadjuvant chemotherapy (NAC)	55	62.5	58	75.3	0.077 a
Neoadjuvant chemoradiotherapy (NACRT)	33	37.5	19	24.7	
Chemotherapy type					
ECF/EOX/ECX	32	36.4	33	42.9	0.357 a
FLOT	24	27.3	24	31.2	
Others-CROSS/CF/Ciscape	32	36.4	20	26	
Chemotherapy completion					
No	9	10.2	18	23.4	0.016 a
Yes	79	89.8	56	72.7	
Missing	0	0	3	3.9	
Clavien–Dindo (CD) excluding CD 5 d					
CD 0–2	68	80	49	64	
CD 3–4	17	20	27	36	0.034 c
Tumour regression grade (TRG)					
All patients					
Responder Mandard 1–3	49	56	35	45	
Non-responder Mandard 4–5	38	43.2	42	55	0.211 c
Missing	1	1.14			
Neoadjuvant chemotherapy					
Responder Mandard 1–3	22	41	20	35	
Non-responder Mandard 4–5	32	59	38	65	0.494 a
Neoadjuvant chemoradiotherapy					
Responder Mandard 1–3	27	82	15	79	
Non-responder Mandard 4–5	6	18	4	21	0.800 a
Anastomotic Leak					
No	83	94.3	71	92.2	0.588 a
Yes	5	5.7	6	7.8	
Pneumonia					
No	74	84.1	62	80.5	0.548 a
Yes	14	15.9	15	19.5	
Post-operative mortality					
Yes	35	39.8	31	40.3	0.878 a
No	51	58	43	55.8	
Missing	2	2.3	3	3.9	
Recurrence					
No	64	72	58	75	
Yes	24	28	18	23	
Missing			1	2	

ASA American Society of Anesthesiologists physical status class, BMI body mass index, SD standard deviation, ECF (cisplatin, epirubicin and fluorouracil), EOX (epirubicin, oxaliplatin and capecitabine), ECX (epirubicin, cisplatin and capecitabine), FLOT (docetaxel–fluorouracil–folinic acid–oxaliplatin), CROSS (carboplatin, paclitaxel, concurrent radiotherapy), CF (5-fluorouracil + cisplatin), Ciscape (cisplatin and capecitabine). a: Chi-square test, b: *t*-test, c: Fisher’s exact test, d: excluding M1 patients.

**Table 4:** Delta VO_2 peak_ (changes in VO_2 peak_ in prehab vs. control group, inclusive of M1 patients and all surgery types).

	Prehabilitation Group (N = 88)	Control Group (N = 77)	*p*-Value for *t*-Test
Baseline VO_2 peak_ (mean (s.d.))	22.19 (5.54)	22.18 (3.89)	
Post-NAT VO_2 peak_(mean (s.d.))	20.92 (3.99)	19.23 (3.25)	
Changes in VO_2 peak_(s.d.)	−1.07 (4.47)	−2.74 (2.91)	0.035

**Table 5:** Delta anaerobic threshold (AT) (changes in AT in prehab vs. control group, inclusive of M1 patients and all surgery types.

	Prehabilitation Group (N = 88)	Control Group (N = 77)	*p*-Value for *t*-Test
Baseline AT (mean (s.d.))	14.04 (3.69)	14.48 (3.24)	
Post-NAT AT (mean (s.d.))	12.81 (2.62)	11.81 (2.68)	
Changes in AT (s.d.)	−0.96 (4.00)	−1.78 (5.72)	0.385

## Data Availability

The data used to support the findings of this study are included within the article. The datasets used and/or analysed during the current study will be available from the corresponding author on reasonable request.
